# Surgical outcome after autologous bone chips replacement in depressed skull fractures: a single center experience

**DOI:** 10.1007/s10143-024-03128-y

**Published:** 2024-12-10

**Authors:** Hossam Elnoamany, Ahmed Mansour, Mazen Lotfy Agour, Mohammed Dorrah, Nour Elnoamany, Anwar Hourieh, Hany Elkholy

**Affiliations:** 1 Department of Neurosurgery, Menofia University, Shibin Elkom, Menofia, 32511 Egypt; 2 Menofia University Hospitals, Shibīn Elkom, Menofia, Egypt; 3 Faculty of Medicine, Menofia University, Shibin Elkom, Menofia, Egypt

**Keywords:** Head injury, Depressed skull fracture, Autologous bone fragments, Aesthetic results, Cranioplasty

## Abstract

**Background:**

Surgery for depressed skull fractures (DSFs) is always faced by multiple challenges including ideal timing, defect reconstruction and complications. Few data are available regarding the aesthetic results and patients’ satisfaction following DSFs management.

**Methods:**

A prospective non-randomized study included 59 traumatic brain injury (TBI) patients surgically treated for DSFs. Depressed bone fragments were elevated and washed with diluted hydrogen peroxide for 15 min then replaced within a net made of vicryl 0 through edges of the galea. Our objective was to evaluate outcome and patients’ satisfaction of using autologous bone fragments for skull defect reconstruction.

**Results:**

The mean Glasgow Coma Scale (GCS) score on admission was 14.51 ± 1.237. The mean age was 16.505 ± 12.426 years. DSFs were of compound type in 81.4% with predominance towards the parietal region 54.2%. Associated intracranial pathologies were found in 39.0% of cases. Mean time to surgery was 5.79 ± 9.982 h. Dura was found torn in 19 cases (32.2%). Postoperative complications were encountered in 5 cases (8.5%). The mean hospital stay was 3.61 ± 3.157 days. 96.6% of cases had good discharge outcome. Factors with significant impact on outcome included; admission GCS score (*P* < 0.001), type of associated pathology (*P* = 0.006), and venous sinus involvement (*P* = 0.003). At the end of follow up, 46 patients (82.5%) were satisfied about the aesthetic results, while 10 patients (17.5%) were not satisfied and 9 of them underwent re-surgery for late cranioplasty.

**Conclusions:**

Using autologous depressed bone chips for skull defect reconstruction can be a safe and feasible surgical technique for TBI patients suffering DSFs with good aesthetic results, high patient satisfaction, decreased need for later cranioplasty and consequently low overall management cost.

## Introduction

Skull fractures can be classified by pattern (linear, comminuted or depressed), by anatomic location (skull vault or base), and by skin integrity (open or closed) [1]. Depressed skull fractures (DSFs) have a consistent association with higher incidence of intracranial lesions, neurological deficits, and unfavorable outcome [2, 3].

Surgical treatment for DSFs is indicated whenever: the bone depression is beyond the inner table of non-depressed skull bones; it is a compound fracture; associated with focal neurological signs; suspected cerebrospinal fluid (CSF) leak; associated with intracranial lesions; or there is cosmetic purpose [4].

Computed tomography (CT) and magnetic resonance imaging (MRI) pave path for significant advancements in understanding brain injuries and surgical techniques. Cranioplasty (CP) following the elevation of depressed bone chips can provide protective, cosmetic and neurological benefits. The appropriate choice of implant, ideal timing, complications, and avoiding reoperation are challenges that neurosurgeons face in managing DSFs [5–7].

To the best of our knowledge, previous studies conducted on DSFs focused on the postoperative outcome, and only a few recent studies analyzed the success and satisfaction rates of using autologous depressed bone fragments for skull defect reconstruction. Hence, in this study we aimed to evaluate different patterns of outcome including (safety, efficacy, complications, patients’ satisfaction and need for late CP) of using autologous bone chips for management of DSFs.

## Methods and materials

### Study design and patients population

A prospective non-randomized case series study that was conducted after approval by the local ethical scientific committee of our institution (IRB approval number: 3-2023.NEUS 2 − 1). This study included 59 traumatic brain injury (TBI) patients diagnosed with DSFs who were surgically treated in our Neurosurgery Department over a period of 9 months started in March 2023 and ended in November 2023. Patients were followed up in our outpatient clinic for 6 months after surgery.

### Eligibility criteria

We included all TBI patients of either sex with no age restrictions presented to our Emergency Department (ED) with DSFs that required surgical intervention. We excluded (1) patients with severe TBI (Glasgow Coma Scale (GCS) score ≤ 8); (2) patients operated elsewhere for DSFs and come for follow up; (3) patients in whom the depressed bones are comminuted or lost; (4) patients with spinal cord injury or any life threatening emergency condition rather than cranial injury; (5) patients didn’t continue for follow up; (6) patients with compound DSF presented more than 24 h after trauma.

### Data collection

Data were collected by data collaborators under the leadership of the investigators in our ED. Taking in mind questionnaires that are in the same field of our study, we designed a questionnaire that was signed by each included patient. Also, a questionnaire for patients’ satisfaction with the aesthetic result of autologous bone chips replacement was designed and filled by each case at the end of follow up.

Data were collected from participants who fulfill our eligibility criteria and in the setting we chose, just after admission to ED, at discharge and 6 months after surgery. Patients had the right to withdraw at any time, even if it happened in the follow up questionnaire of our study. Data collection was done by the same data collectors for the same patients.

### Management

All included patients were treated according to the management protocol endorsed by our neurosurgery department, and in agreement with the management guide lines reported in literature which included the following: (1) primary survey to ensure patent airway, intact breathing ability, and adequate blood circulation; (2) complete history: age; sex; mood of trauma (road traffic accident (RTA), fall from height (FFH), assault or direct trauma; clinical presentations (loss of consciousness (LOC), headache, vomiting, amnesia, seizures); (3) thorough general examination to detect other traumatic injuries and co-morbidities; (4) full neurological assessment (GCS score; severity of head injury including mild (GCS 13–15), moderate (GCS 9–12) or severe (GCS 4–8); pupillary responses and neurological deficits); (5) type of skull fracture either a compound DSF (opened skin wound directly over the fracture) or a simple DSF (with either closed skin or with opened skin wound away from the fracture); (6) routine laboratory investigations (complete blood picture, coagulation profile, liver and kidney function tests, random blood sugar, virology markers; (7) CT scan of the brain and skull bones to detect (side, location, size of the skull defect and associated intracranial pathologies).

Dehydrating measures, cerebro-protective agents were given in certain cases. Broad spectrum antibiotics and anti-convulsive drugs were started preoperatively for all cases. Surgical decision was taken by the neurosurgeon consultant on-duty. All surgical lesions were treated under general anesthesia (GA) with standard surgical procedures.

### Surgical approach

**Fig. 1:**
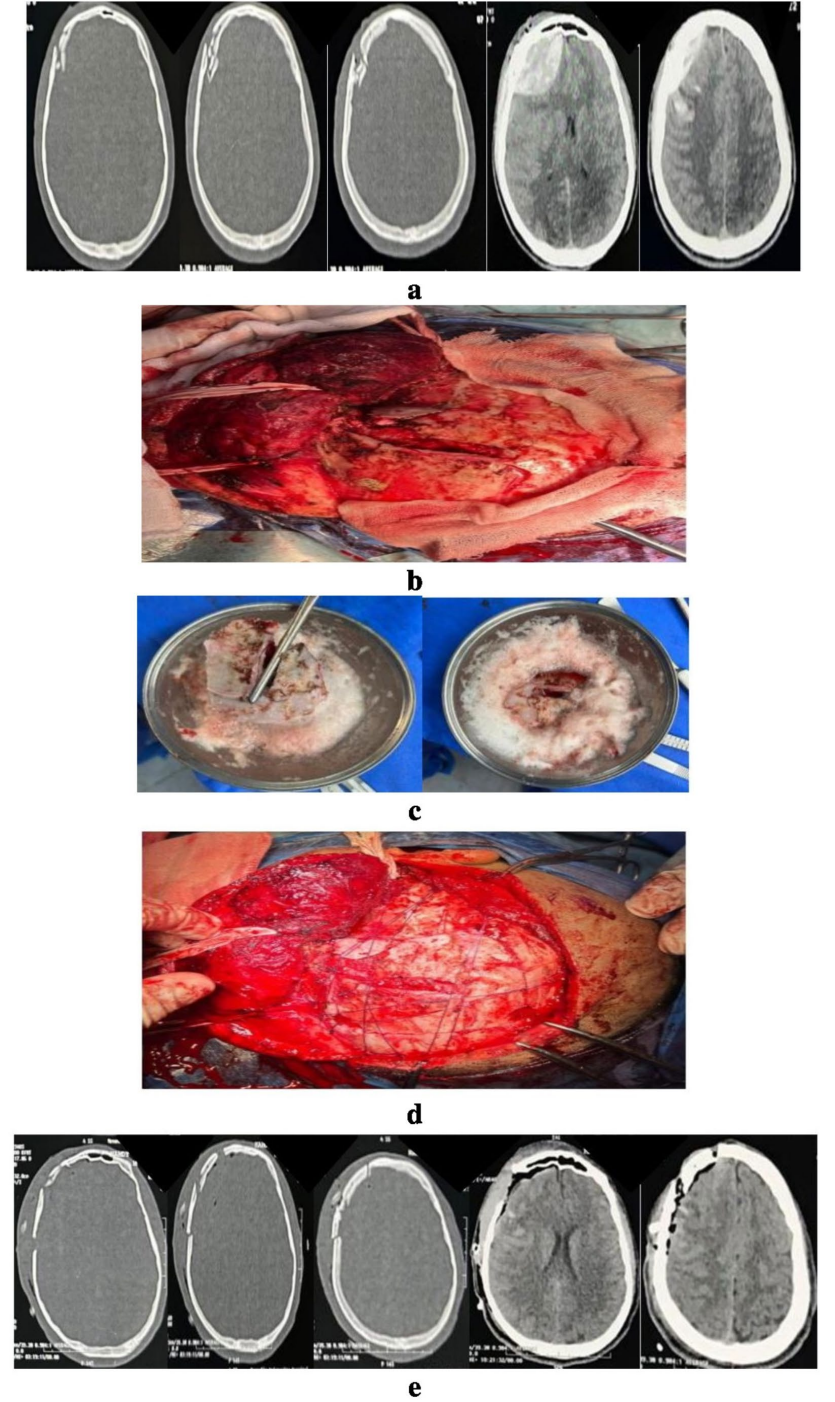
Young adult male patient arrived to our ER after RTA with GCS 12/15. (**a**) Preoperative CT scans showing right frontal DSF with underling EDH. (**b**) Intraoperative image showing the skull defect and depressed bone fragments. (**c**) Intraoperative image showing depressed bone fragments while being washed of in diluted hydrogen peroxide. (**d**) Intraoperative image showing defect reconstruction using autologous bone fragments fixed with a net of vicryl. (**e**) Postoperative CT scans showing good reconstruction of the skull defect

The surgical approach was either through the same wound or with slight expansion. The elevated depressed bone fragments were washed with diluted hydrogen peroxide for 15 min and the surgical field was also irrigated of with diluted hydrogen peroxide. Actually in our emergency operating room we did not have the facilities of mini-plates to fix bone fragments together. Also, we consider that making holes in the sitting of multiple bone chips can be time consuming. Hence, we used vicryl 0 to make a net through edges of the galea then bone fragments were replaced and fixed using this net. Figure 1 illustrates the intraoperative images using our management protocol for a case suffering DSF combined with extradural hematoma (EDH). Lastly, debridement of the wound margins was done followed by skin closure with subgaleal drain.

**Fig. 2:**
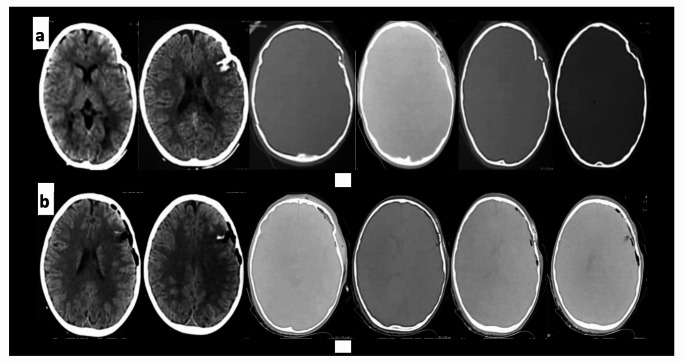
Young adult male patient with history of assault came to our ER with GCS 15/15, complaining from headache and vomiting. (**a**) Preoperative CT scans showing left fronto-parietal DSF with underling small cerebral contusion. (**b**) Postoperative CT showing good reconstruction of the skull defect using autologous bone fragments

Craniotomy flab was done in cases with concomitant intracranial pathology that required surgical intervention. Figure 2 illustrates the pre and postoperative CT images for one of our included cases.

Recorded operative notes included: operative time; intraoperative blood loss; the need for blood transfusion. A broad spectrum antibiotic - Third generation cephalosporins (Ceftriaxone: 30 mg per kg in pediatrics and 2 g in adults) - was administered intravenously immediately before the skin incision and again 12 and 24 h after surgery. Then, patients with opened skin wound (compound DSFs) were maintained on oral antibiotics (first or second generation cephalosporins) for 2 weeks.

Postoperatively: drain removal was after 24 h; patients were kept under close observation with repeated neurological examinations. For all patients, postoperative CT scan was performed in the first 24 h after surgery. After discharge, follow up was conducted for 6 months; all cases were instructed to avoid activities that may expose them to repeated head trauma and all were advised to wear protective head helmet when returning to their daily activities.

### Outcome measures

The primary end point was the discharge outcome. The secondary endpoint was the patient’s satisfaction and the need for delayed cranioplasty after 6 months. Table 1 demonstrates the questionnaire for patients’ satisfaction with the aesthetic result of autologous bone chips replacement.

**Table 1:** Questionnaire for patients’ satisfaction with the aesthetic result about the use of autologous bone chips replacement

Question	Answer
1. Patients’ estimate for the size of postoperative residual bone defect	a. Small b. Medium c. Large
2. Patients’ degree of satisfaction with the aesthetic result about using autologous bone chips replacement.	a. Don’t accept b. Not satisfied c. Satisfied d. Very satisfied
3. Reason of dissatisfaction.	a. Residual defect b. Bulges c. Scars
4. Complications after autologous bone replacement?	a. Yes b. No

**Notes**: these points were assessed at the end of follow up period (6 months after surgery) for the 57 cases who continued for follow up (2 cases were excluded because they were expired during admission

Outcome measures included: (1) intraoperative success rate of using autologous depressed bone fragments for reconstructing the skull defect; (2) postoperative complications, morbidity and mortality rates; (3) discharge outcome; (4) patients’ satisfaction at 6 months after surgery based on the included questionnaire; (6) the need for late cranioplasty.

The Extended Glasgow Outcome Scale (GOSE) score was used to measure the surgical outcome at discharge. Patients who had moderate disability or good recovery (GOSE score from 5 to 8) were included together in the good outcome group. Patients who were severely disabled, vegetative or died (GOSE score from 1 to 4) were included together in the poor outcome group.

### Statistical analysis

To tabulate and statistically analyze the results, SPSS V.22 (IBM Corporation, 1 Orchard Rd, Armonk, NY 10504, USA), and Microsoft Excel 2010 (Microsoft Corporation, One Microsoft Way Redmond, WA 98052 − 6399 USA) were used. The descriptive statistics included mean (x), median, and standard deviation (SD). The count data were expressed as the rate and analyzed using the chi-square test (X^2^). *P* value ≤ 0.05 was considered statistically significant.

## Results

During the study period, a total of 68 TBI patients with DSFs were operated upon in our neurosurgery department. Based on our eligibility criteria, 59 patients fulfilled our inclusion criteria and were actively included.

Among our included cases, (89.8%) had mild TBI and the remaining (10.2%) had moderate TBI. The mean age was (16.505 ± 12.42) years with range from 6 months to 59 years. The vast majority of patients 41 cases (69.5%) were ≤ 20 years and 18 patients (30.5%) were > 20 years. Males constituted the main bulk of study population (78%) and female represented only (22%). RTA was the most frequent mood of trauma (35.6%) followed by assault (23.7%), direct trauma (23.47%) then FFH (16.9%).

The mean GCS score on admission was 14.51 ± 1.237 (range from 9 to 15). The mean time between trauma and surgery was 5.79 ± 9.982 h. None of the included cases had history of previous cranial surgery or significant head trauma. Headache was the most common presentation among included cases (91.5%); the Visual Analogue Scale (VAS) score for preoperative headache was 5.58 ± 3.035. Table 2 demonstrates the general criteria of the included patients.

**Table 2:** General and Radiological criteria among included patients:

Parameter	Frequency	%
**Socioeconomic State**		
Low	21	35.6%
Middle	27	45.8%
High	11	18.6%
**Time from Trauma to Surgery**		
< 6 h	50	84.7%
6–24 h	6	10.2%
> 24 h	3	5.1%
**Clinical Presentations**		
Headache	54	91.5%
Vomiting	24	40.7%
LOC	6	10.2%
Amnesia	5	8.5%
Convulsions	3	5.1%
Motor Deficit	5	8.5%
**Side of DSFs**		
Left	34	57.6%
Right	22	37.3%
Midline	3	5.1%
**Location of DSFs**		
Parietal	32	54.2%
Frontal	20	33.9%
Fronto-parietal	3	5.1%
Temporo-parietal	2	3.4%
Occipital	2	3.4%
**Associated Pathologies**		
EDH	10	16.9%
Contusion	6	10.2%
Pneumocephalus	3	5.1%
EDH + Contusions	2	3.4%
SAH	2	3.4%
None	36	61.0%
**Venous Sinus involvement**		
SSS	5	8.5%
Transverse Sinus	1	1.7%
None	53	89.8%

**Notes**: DSFs: depressed skull fractures; RTA: road traffic accident; FFH: fall from height; LOC: loss of consciousness; EDH: extradural hematoma; SAH: subarachnoid hemorrhage; SSS: superior sagittal sinus

In the present series, all cases were diagnosed using CT scan of the brain and skull bones; 2 cases (3.4%) required MRI brain for more evaluation. The majority of cases (81.4%) had compound (open) type of DSFs, and the remaining (18.6%) had simple (close) type. The commonest location for DSFs was the parietal skull region (54.2%). Concomitant intracranial pathologies were encountered in (39.0%) of cases with EDH and cerebral contusions were the most frequent.

The mean operative time was 80.81 ± 20.796 min. Surgical elevation of depressed bone chips with removal of in-driven bone fragments was done in all of cases. Twelve cases (20.3%) required craniotomy flap for concomitant intracranial lesions. Dura was found torn in 19 cases (32.2%); it was repaired with direct sutures in 16 cases and with dural graft in 3 cases.

The mean postoperative GCS score was 14.81 ± 9.15 with significant improvement (*p* = 0.001). Postoperative complications were encountered in 5 cases (8.5%) and included seizures (3.4%), wound infection (3.4%), CSF leakage (3.4%) and osteomyelitis (1.7%). Only two cases (3.4%) were expired. Postoperatively, the VAS score for headache was significantly improved to 2.42 ± 1.610 at discharge and to 0.68 ± 1.706 after 6 months (*p* < 0.001). One case (1.7%) required re-surgery during the period of admission because of wound infection and osteomyelitis. Among the 5 cases with preoperative neurological deficit, 2 cases (3.4%) were improved and 3 cases (5.1%) remained as preoperative. The mean duration of hospital stay was 3.61 ± 3.157 days ranged from (1–24) days. The mean GOSE score at discharge was 7.20 ± 1.297. As demonstrated in Table 3, the only factor with significant impact on discharge outcome was the TBI grade (*P* = 0.000). The majority of cases (96.6%) showed favorable discharge outcome.

**Table 3:** Factors affecting the discharge outcome:

Preoperative Factor		Discharge Outcome		*P* value
		Good	Poor	
TBI Grade	Mild	53	0	0.000*
	Moderate	4	2	
Mode of Trauma	RTA	20	1	0.667
	FFH	10	0	
	Assault	13	1	
	Direct	14	0	
Time from trauma to surgery	< 6 h	48	2	0.830
	6–24 h	6	0	
	> 6 h	3	0	
Associated Pathology	Yes	21	2	0.072
	No	36	0	
Fracture Type	Simple	10	1	0.247
	Compound	47	1	
Defect Size	Small	32	2	0.467
	Large	9	0	

Notes: TBI: Traumatic Brain Injury; RTA: road traffic accident; FFH: fall from height; h: hour; * Statistically Significant

Patients were followed up for 6 months; Table 4 demonstrates the outcome parameters and estimated satisfaction among our included cases at the end of follow up (excluding the 2 expired cases). Table 5 demonstrates factors affecting the need for cranioplasty after 6 months.

**Table 4:** Outcome after 6 months:

Outcome parameter	Frequency	%
**Patient Satisfaction with the aesthetic result**		
Don’t accept	3	5.2%
Not satisfied	7	12.3%
Satisfied	16	28.1%
Very satisfied	31	54.4%
**Patients’ estimate for the size of residual bone defect**		
Small	33	57.9%
Medium	15	26.3%
Large	9	15.8%
**Reason of dissatisfaction.**		
Residual defect	9	90%
Scars	1	10%
Bulges	0	0
**Re-surgery for Cranioplasty**		
Yes	9	15.3%
No	50	84.7%
**Neurological Deficit**		
Same as preoperative	2	3.4%
Improved	3	5.1%

**Notes**: The outcome after 6 months was estimated among the 57 cases (excluding the 2 expired cases)

**Table 5:** Factors affecting the need for cranioplasty after 6 months:

Preoperative Factor		Delayed Cranioplasty (*n*)	*P* value
TBI Grade	Mild	7	0.194
	Moderate	2	
Mode of Trauma	RTA	3	0.290
	FFH	0	
	Assault	2	
	Direct	4	
Time from trauma to surgery	< 6 h	8	0.752
	6–24 h	1	
	> 6 h	0	
Associated Pathology	Yes	3	0.706
	No	6	
Defect Size	Small	0	0.000*
	Large	9	

Notes: TBI: Traumatic Brain Injury; RTA: road traffic accident; FFH: fall from height; h: hour; * Statistically Significant

## Discussion

Unfortunately, the incidence of TBI is steadily increasing not only in developing countries but also in the developed countries. TBI contributes to a high mortality and morbidity among trauma patients; hence concerns always arise for better management hoping a more satisfactory outcome.

Among studied cases, the mean age was (16.505 ± 12.426) years and 35.6% of patients were ≤ 10 years old. The incidence of DSFs was higher in males (78%). These results are compatible with previous studies [8, 9]. In contrary to our result, in **Manne S et al.** [10] and **Satardey et al.** [11] stated that the mean patients’ age was (27.9 and 37.6 years respectively).

We found that, the most common mode of trauma was RTA (35.6%). **Manne S et al.** [10] and **Satardey et al.** [11] reported similar results while in **Heary and associates** [12] study, the incidence of DSFs was roughly equal after RTAs and assault and in **Al-Derazi et al.** [13] study, accidental fall of heavy object on the head mainly during industrial work or building constructions was the cause of injury in (30%) of patients.

Compound DSFs result in communication between the external environment and the cranial cavity, it may be clean or contaminated/dirty [14]. The majority of our cases (81.4%) had an open type DSFs; this finding is in consonance with most of previous studies [9–11]. The commonest locations for DSFs were the parietal skull region (54.2%) followed by the frontal region (33.9%). The fronto-parietal region of skull is thin and so can be the most prone location to an assailant’s attack [10–14].

The force severe to cause skull fracture usually expends itself in the skull or may spread producing brain damage as well. Depressed bone fragments may cause brain lacerations or damage of the blood vessels [4, 15]. In the present study, (39.0%) of cases had associated post-traumatic intracranial pathologies with EDH was the most common (16.9%) followed by cerebral contusions (10.2%). Most of previous studies reported frequent association of DSFs with underling intracranial hemorrhage or contusions [9–11, 16].

Compound DSFs are considered surgical emergencies, prompt treatment is mandatory to avoid complications as posttraumatic seizure, intracranial infection and osteomyelitis [14, 15]. In our case series, the 59 included head trauma patients underwent surgical treatment for DSFs with simultaneous replacement of depressed autologous bone chips using our management protocol (washing the bone fragments with diluted hydrogen peroxide for 15 min; irrigation of the surgical field with diluted hydrogen peroxide; and fixing bone fragments using a net made of vicryl sutures through the galea). Using broad spectrum antibiotics can be of great value to minimize the infection risk. Application of an adapted antibiotic prophylaxis to all patients undergoing the same intervention guided by empirical data covering for the expected pathogens and avoiding potential first-line therapeutics may possibly be a better choice [17, 18]. So, in our series third generation cephalosporins was administered intravenously immediately before the skin incision and again 12 and 24 h after surgery. Then, patients with opened skin wound (compound DSFs) were maintained on oral antibiotics (first or second generation cephalosporins) for 2 weeks.

Intra-operatively, we found dural tear in 19 cases (32.2%); it was repaired with direct sutures in 16 cases and with dural graft in 3 cases. **Hossain and associates** [4], reported dural tear in 25% of cases, and **Satardey et al.** [11] study reported dural tear in 14% of cases.

In our case series, 2 cases (3.4%) developed postoperative wound infection. Staphylococcus aureus was the identified causative organisms in the 2 cases. The results of culture and sensitivity did not guide antibiotic treatment, and we started empiric broad-spectrum antibiotic regimen with vancomycin (15 mg/kg IV every 12 h) plus a third a generation cephalosporin (ceftriaxone 2 g IV daily). One of the two cases showed good response to the antibiotic treatment while the other developed osteomyelitis and underwent re-surgery for wound debridement and removal of bone fragments, later this case was expired. In the series of **Sidram V and associates** [9], wound infection was observed in (11.3%) of cases and it was significantly associated with higher incidence of persistent neurological deficit, late epilepsy and death.

In literature, there is great controversy in regards to the prophylactic use of preoperative antibiotics and anti-epileptics in head injury patients [3, 11]. Worthy mentioned that, the lower rate of postoperative wound infection encountered in our series can be attributed to multiple factors including that: all cases were operated within the first 24 h after trauma; broad spectrum antibiotics were initiated preoperatively and continued for 2 weeks in cases with open DSFs; intraoperative washing of bone fragments with hydrogen peroxide for 15 min together with debridement of wound margins. In **Jennett B et al.** [19] study, operative debridement reduced the incidence of infection to (4.6%) in their series.

Other encountered complications included; postoperative seizures (3.4%), CSF leak (3.4%) and osteomyelitis (1.7%). In **Sidram V et al.** [9] series, meningitis was reported in (4.5%), CSF leak in (9.1%), and pseudomeningocele in (2.1%).

In our case series, 57 cases (96.6%) had good discharge outcome while 2 cases (3.4%) showed poor discharge outcome and were expired. The grade of TBI was the only preoperative factor that showed significant impact on discharge outcome; where the 2 cases with poor discharge outcome had moderate TBI and low admission GCS. There was no statistically significant association (*P* > 0.05) between discharge outcome and either patient’s age, gender, socioeconomic state, fracture type (simple or compound) or fracture location. Similar results were reported in **Manne S et al.** [10] study. In contrary to our result, **Satardey et al.** [11] and **Jagger et al.** [20] concluded that outcome became worse with increased age.

**Satardey and associates** [11] study reported that poor outcomes had statistically significant relation with fracture type (simple fractures had better outcomes in their study). This was not observed in our study; where among the 2 cases with poor outcome one had simple and the other had compound DSF.

In our series, the mode of trauma had no significant impact on discharge outcome (*P* = 0.667). In contrary to this **Manne S and associates** [10] reported significantly poor outcome in the RTA group when compared with the non‑RTA group (*P* < 0.05).

In the current study, admission GSC score and the grade of TBI showed significant impact (*P* < 0.001) on discharge outcome. In literature, strong correlation between GCS on admission and final outcome in TBI patients was reported where patients with GCS score of 13–15 on admission fared well with better long‑term outcome as against those with low GCS score [4, 10, 11].

There is a consistent association between the presence of cranial fracture and higher incidence of intracranial lesions, neurological deficit, and poor outcome [2, 3]. In our case series, the type of associated pathology (*p* = 0.006); and involvement of the superior sagittal sinus (SSS) (*p* = 0.003) showed significant association with discharge outcome. Similar to our result, **Manne S and associates** [10] reported that most cases with poor outcome had concomitant cerebral contusions with a statistically significant association (*P* < 0.05).

In our series, 2 cases (3.4%) presented with compound DSFs with underling pathology and had low preoperative GCS score; unfortunately the two cases were expired. Close to our result, **Manne S et al.** [10] reported 3 expired cases (2%). In literature, the mortality rate is between 1.4 and 19% [21, 22].

### Outcome after 6 months

None of our cases developed late postoperative infection and the VAS score for postoperative headache was significantly decreased from (5.58 ± 3.035) preoperatively to (0.68 ± 1.706) after 6 months (*p* < 0.001).

Worthy noted that, the literature has limited data addressing the cosmetic outcome and the aesthetic results following DSFs management. This might be a result of the fact that neurological disability and functional outcome following TBI are much more important than the cosmetic results [23].

In our series, the aesthetic results were significantly high among included cases. Of all 57 patients who continue for follow up, 46 patients (82.5%) were found to be satisfied, and 10 patients (17.5%) were not satisfied. Follow up CT scans documented that, there was no bone migration or displacement in any case. The size of bone defect estimated by participants was as small in 33 cases (57.9%), medium in 15 cases (26.3%), and large in 9 cases (15.8%). Among the 10 cases who were not satisfied, 9 patients (90%) referred their dissatisfaction to the residual defect while one case (10%) referred it to the wound scar. Notably, the grade of satisfaction did not change over time in all cases from the time of discharge till the end of follow up. A study done by **César A et al.** [24] included 29 operated patients for CP using autologous bone after decompressive craniectomy, 25 patients (86.2%) were satisfied with the aesthetic result while the remaining 4 patients (13.8%) were not satisfied.

By the end of the 6 month follow up period, only 9 cases (15.3%) underwent re-surgery for late CP. This result magnifies the value of autologous bone chips replacement in saving the patient from going for second surgery with its different patterns of loads and costs.

No doubt that, skull defect reconstruction using autologous bone chips is a low cost technique in comparison with synthetic materials used for CP. In a recent study by **Findlay M and associates** [25] comparing the cost of autologous versus non-autologous cranioplasty, they reported that costs of custom cranioplasty were significantly more expensive than autologous grafts (*p* < 0.01). Cost subcategories with statistically significant differences between groups included implant costs (89.2% versus 12.2%, *p* < 0.01) and pharmacy costs (3.1% versus 4.4%, *p* = 0.03). **Lethaus et al.** [26] found that patient-specific implants (PEEK or titanium) were more costly than autologous grafts in the initial surgery. **Binhammer and associates** [27] reported in their analysis that, titanium mesh grafts were more cost-effective than autologous grafts in the setting of smaller cranial defects.

### Study limitations

The limitations of the current study may come from being a single center experience. Although there was a standard protocol for the surgical management of DSFs, operations were performed by different surgeons. In regards to our follow up (6 months after surgery) that may seem to be insufficient to rule out bone resorption and need for wound reopening and cranioplasty; our participants were regularly followed in the outpatient clinic and follow up CT scans documented that, there was no bone migration or displacement in any case. However, we believe that this baseline information can be important and can provide a basis for comparison for future trials.

## Conclusions

Using autologous depressed bone chips for skull defect reconstruction can be a safe and feasible surgical technique for TBI patients with DSFs. Washing the bone fragments with diluted hydrogen peroxide for 15 min, irrigation of the surgical field with diluted hydrogen peroxide, and fixing bone fragments using a net made of vicryl sutures through the galea was associated with good aesthetic results, high patient satisfaction, decreased need for later cranioplasty and consequently low overall management cost.

## Data Availability

Data is provided within the related data files.

## Abbreviations

DSFs: Depressed skull fractures
CSF: Cerebrospinal fluid
CT: Computed tomography
MRI: Magnetic resonance imaging
CP: Cranioplasty
TBI: Traumatic brain injury
ED: Emergency Department
GCS: Glasgow Coma Scale
RTA: Road traffic accident
FFH: Fall from height
GA: General Anesthesia
GOSE: Extended Glasgow Outcome Scale
VAS: Visual Analogue Scale
EDH: Extradural Hematoma
SAH: Subarachnoid Hemorrhage
SSS: Superior Sagittal Sinus
